# Centrosome aberrations and chromosome instability contribute to tumorigenesis and intra-tumor heterogeneity

**DOI:** 10.20517/2394-4722.2018.24

**Published:** 2018-08-07

**Authors:** Shirley Jusino, Fabiola M. Fernández-Padín, Harold I. Saavedra

**Affiliations:** Basic Sciences Department, Division of Pharmacology and Toxicology, Ponce Health Sciences University, Ponce Research Institute, Ponce, PR 00732, USA.

**Keywords:** Centrosome, chromosome instability, intra-tumor heterogeneity, single-cell sequencing

## Abstract

Centrosomes serve as the major microtubule organizing centers in cells and thereby contribute to cell shape, polarity, and motility. Also, centrosomes ensure equal chromosome segregation during mitosis. Centrosome aberrations arise when the centrosome cycle is deregulated, or as a result of cytokinesis failure. A long-standing postulate is that centrosome aberrations are involved in the initiation and progression of cancer. However, this notion has been a subject of controversy because until recently the relationship has been correlative. Recently, it was shown that numerical or structural centrosome aberrations can initiate tumors in certain tissues in mice, as well as invasion. Particularly, we will focus on centrosome amplification and chromosome instability as drivers of intra-tumor heterogeneity and their consequences in cancer. We will also discuss briefly the controversies surrounding this theory to highlight the fact that the role of both centrosome amplification and chromosome instability in cancer is highly context-dependent. Further, we will discuss single-cell sequencing as a novel technique to understand intra-tumor heterogeneity and some therapeutic approaches to target chromosome instability.

## INTRODUCTION

Intra-tumor heterogeneity is a cancer hallmark that is characterized by the presence of different cell subpopulations within the same tumor^[1,2]^. These cell sub-populations foster tumor adaptation and evolution that hinders cancer treatment and leads to tumor recurrence and metastasis^[3,4]^. Therefore, despite the great conceptual and technological advancements in cancer research, recurrence and metastasis remain a key clinical challenge, making cancer the second leading cause of death in the United States. In this review, we discuss some classical experiments that have enlightened us as to our understanding toward cell cycle and centrosome regulation in order to understand how this modulates cancer initiation, maintenance, progression, and causes intra-tumor heterogeneity. We also discuss other causes of intra-tumor heterogeneity, such as the cancer stem cell theory. We also discuss the single-cell sequencing technique, as a novel technique to understand intra-tumor heterogeneity and relevant therapeutic targets that may aid our understanding of cancer and envision a more effective treatment.

## THE CAUSES AND CONSEQUENCES OF INTRA-TUMOR HETEROGENEITY IN CANCER

Intra-tumor heterogeneity describes the existence of different genetic subpopulations of cells in a given primary tumor^[1]^. Genetic heterogeneity is studied to determine the transcriptional expression, copy number or mutational/polymorphic status of genes within a tumor to provide an overall tumor genetic composition and determine the best treatment option for patients^[5]^, which is the basis for personalized medicine. Genetic, epigenetic, and metabolic changes are important contributors to tumor formation and progression^[5]^. Cancer stem cells, genetic and epigenetic alterations, copy number variation (CMV), single nucleotide variants (SNV), aneuploidy, genome duplication, and chromosome instability can initiate and sustain cancer progression and genetic heterogeneity. Intra-tumor heterogeneity supports the theory of clonal evolution that has been forced by selective pressures such as those exerted by chemotherapy or radiotherapy.

It is generally accepted that all cancer types display some degree of intratumoral heterogeneity, with thyroid and prostate cancers showing less heterogeneity, and cancers that include lung, stomach, glioblastomas and melanomas displaying a high degree of intratumoral heterogeneity^[2]^. In fact, transcriptomic and genomic profiling of multi-spatial biopsies of glioblastomas, medulloblastomas and renal cell carcinomas demonstrated that cells within a single tumor were rarely clonal, thus explaining single-agent therapy failure in cancers^[6]^. Genetic heterogeneity determines the fate of metastasis, with highly heterogeneous cancers such as colon displaying highly heterogeneous metastases within the same patient^[7]^. On the other hand, many high-grade serous ovarian cancers of patients with metastases are clonal, and most metastases originate from one clone^[8]^. Breast cancers are excellent examples of the role played by genetic heterogeneity in survival outcomes of affected patients^[1]^. Breast cancers are classified using mRNA expression microarrays and/or with several pathological markers, including the epidermal growth factor 2 (Her2), the estrogen receptor (ER), or the progesterone receptor (PR). The classification includes Luminal A (ER^+^PR^+^Her2^−^), Luminal B (ER^+^PR^+^ and Her2^+^ or Her2^−^), Her2^+^ (ER^−^PR^−^Her2^+^) and basal (which includes 76% triple-negative breast tumors, ER^−^PR^−^Her2^−^)^[9]^. Luminal A breast cancer patients show the best survival of all breast cancer patients, followed by Luminal B, Her2^+^ and basal^[10,11]^. More recent studies show that hormone receptor-negative breast tumors (Her2^+^ and basal) display more chromosome instability and centrosome amplification (defined as the acquisition of three or more centrosomes that promote the formation of a bipolar mitotic spindle and equal segregation of chromosomes following mitosis) than luminal subtypes^[12,13]^. Also, Her2^+^ and triple-negative basal breast cancer patients that initially respond to chemotherapy tend to relapse more readily than luminal breast cancer patients if residual disease remains^[14]^. Molecular subtypes also determine the preferred metastatic sites of breast cancer cells, since Luminal subtypes are more likely to invade the bone, and basal subtypes are more likely to invade into the lung^[15]^. The differences in survival outcomes between luminal and hormone receptor-negative breast cancers can be explained by the plethora of treatments available to treat luminal patients (including tamoxifen, Cdk4/Cdk6 and aromatase inhibitors). Nevertheless, the differences in survival can only be partly explained by differences in treatments available, since similar treatments are available for Luminal A and Luminal B breast cancers, and yet Luminal B breast cancers have poorer survival^[16]^. We speculate that the higher relapse rates are due to the close relationship between aneuploidy, chromosome instability, and chemotherapy resistance^[17,18]^.

Intra-tumor heterogeneity origins can be explained by two theories: clonal evolution and stem cells origin. The first theory, clonal evolution, proposes that intra-tumor heterogeneity arises in response to tumor cell adaptation^[1]^. In this model, the existence of different genetic subpopulations of cells can be due to external pressures that drive the evolution of a tumor following the Darwinian evolutionary principles^[19]^. This theory was first described in 1976 by Peter Nowell, who described cancer progression as an evolutionary process driven by multiple somatic mutations, giving rise to uncontrolled growth and adaptation to the environment^[19,20]^. Then, Loeb proposed that this evolutionary process could be accelerated by a mutator phenotype initially caused by a mutation in genes that control genetic stability^[21]^. Many mouse models have given support to the evidence of such mechanism in mouse models, including experiments done by Fukasawa *et al*.^[22]^, who demonstrated using young mice harboring a genetic knockout of p53 frequent chromosome instability, aneuploidy, and centrosome amplification that preceded tumorigenesis. Other altered tumor suppressors that allow genomic instability include Brca1 and Brca2^[23,24]^. Oncogenes that can cause genetic instability include K-Ras^G12D^, v-Ras, H-Ras^G12V^ and c-Myc^[25–29]^. More recent data by the Pellman group has shown that evolution can also occur from single, catastrophic events^[30,31]^. One of such mechanisms is known as chromothripsis, which is caused by the fragmentation and rearrangements of whole chromosomes contained in micronuclei (defined as missegregated whole chromosomes)^[31]^. Interestingly, centrosome amplification and failure of the spindle assembly checkpoint frequently cause whole chromosome losses^[26,27,32–35]^, implying that they may represent primary causes of these catastrophic events. Genetic mutations not only drive cancer initiation and progression but can sustain cancer cell survival by modulating the metabolism that supplies the high demand of building blocks required by cancer cells. For example, it has been reported that the transcription factors p53, c-Myc, and HIF can induce the expression and activity of glucose transporters involved in glycolysis and the hexose monophosphate shunt to fuel the TCA cycle^[36]^. Moreover, fatty acid β-oxidation is expressed differently in glioblastoma subtypes; this generates a different response to drug treatment and leads to lipid mobilization to generate more energetic compounds and building block for cancer development and progression^[37]^. This adaptation to the environment does not only create an effect in the microenvironment surroundings but also alters the response to therapy by creating cells resistant to chemotherapy.

The second theory, the cancer stem cell (CSC), states that the self-renewal capacity of a stem cell leads to intra-tumor heterogeneity^[1]^. This theory does not take in consideration aberrant genetic errors that may confer genetic advantages to the tumor as the clonal evolution theory does. The presence of CSCs was first observed in chronic myeloid leukemia and mouse models^[19]^. Furthermore, a study done in mice that were injected with breast cancer cells demonstrated the presence of a small subset of cells that displayed the cell surface marker of stem cells, CD44^+^CD24^−/low[38]^. Another tenet of the CSC theory is that tissue-specific stem cells may arise from the accumulation of mutations over time that can initiate tumorigenesis (local or distant), and then become CSC^[39]^. For metastasis to occur, the cancer cells from a primary tumor need to detach, invade the vascular or lymphatic tissue, extravasate, and then proliferate by recruiting surrounding vasculature to grow at a distant site. CSC has been implicated in metastasis through epithelial to mesenchymal transition (EMT), a precursor of metastasis^[40]^. CSC gives origin to the generation of circulating tumor cells (CTCs), defined as rare (1 to 10^6^) cancer cells that circulate in the peripheral blood^[39,41]^ and colonize adjacent tissues; thus contributing to tumor progression. External pressures create a microenvironment that changes the phenotypic and behavioral development of a tumor. This reasoning provides an initial explanation of drug resistance and metastasis initiation between patients with the same type of cancer^[5,39]^. The external pressures can be inflammatory responses, radiotherapy, or cytotoxic chemotherapy^[19,42,43]^. The microenvironment surrounding a tumor can also influence tumor fate. In a recent example, the genetic ablation of the E2F3 transcription factor in macrophages suppresses mammary tumor metastasis into the lungs, but not mammary tumor growth, suggesting that proper macrophage functions and specific microenvironments maintain specific cancer cell functions^[44]^.

## SINGLE-CELL SEQUENCING: A PROMISING TOOL FOR DECIPHERING TUMOR HETEROGENEITY

We discussed in the previous section that cancer stem cells, and changes in genetic and metabolic pathways in whole populations and single cells triggered by chromosome instability generate heterogeneity in cancer cell subpopulations. Even then, these cancer-cell subpopulations are limited in their functionality by distinct microenvironments or physical barriers, and tumor cells adapt to overcome these barriers. This confers adaptive tumor features and generates CTCs. Due to their critical role in intra-tumor heterogeneity, CTCs are well studied by single-cell sequencing. CTCs are found as clusters that reflect the intra-tumor heterogeneity and the potential capacity to initiate metastasis. Alternatively, CTCs can differentiate into different single cells from the initial tumor, thus increasing intra-tumor heterogeneity. Therefore, CTCs can serve as a diagnostic and evolutionary component to a better-targeted therapy^[45–48]^. The most recent technique to study intra-tumor heterogeneity is single-cell sequencing (SCS). SCS is based on the principles that govern the next generation sequencing (NGS) technique. However, SCS is more informative than NGS because it reveals information from a single cell instead of making a pool of several cells that may have a heterogeneous genome and thus affect the results. The SCS procedure can be divided into two stages: single cell isolation and cell genomic profiling. Single cells can be obtained by the use of fluorescence-activated cell sorting (FACS)^[49]^, laser-capture microdissection (LCM)^[47]^, and micromanipulation^[49]^. Out of these, FACS appears to be the most efficient and easier to perform. After obtaining the single cell, single-cell genomic sequencing or single-cell transcriptomic sequencing can be done.

Single-cell genomic sequencing or single nuclear genome sequencing is useful to study mutations, single nucleotide variations, and indels (insertion and deletions)^[50]^. Multiple methods of SCS for single nuclear genome have been designed [Table 1]. One of such variants is the DOP-PCR, in which the amplification of the sequences is started with primers that in the 5’−3’ ends have six possible ACTG combinations, which allow the hybridization of the template with the single cell DNA. This amplification of the sequences generates a database that is used to assess copy number assessment^[39,41,51]^. Another type of DNA sequencing of single cells is the multiple displacement amplification (MDA). This technique is characterized by not having a PCR phase amplification; instead denaturalized DNA from single cells are exposed to anneal with hexamer primers, synthesizing new DNA strands^[52]^. This type of sequencing is a better tool to detect mutations in the DNA strands. Another is the multiple annealing and looping-based amplification cycles (MALBAC) that amplify the original single cell DNA strand^[51]^. Creating a database that is useful for the detection of copy number variants (CNV)^[53]^. An aspect that differentiates all of these types of SCS is the generation of artifacts, false positive and false negative results that can affect the application of the proper algorithm to determine if the changes are significant of the population heterogeneity at the level of single nucleotide variants (SNV).

On the other hand, single-cell transcriptomic sequencing or whole transcriptome sequencing can be used to study the genetic network regulation in a certain cell subpopulation. Also, it can be useful to detect alternative splice sites, novel exons, retained introns, coding RNAs, and non-coding RNAs, among others^[39,41,50]^. Most of the sequencing protocols in cancer research use the whole transcriptome amplification (WTA). WTA uses reverse transcriptase to transform mRNA to cDNA via PCR amplification. This method was first used by Tang and colleagues^[56]^, and they used an oligo-dT primer at 5’ and in the 3’ they added a poly-A tail in the cDNA, generating data to detect alternative splice sites in the mRNA, generation of novel exons in the CTCs and genetic variants in the strand. Two main variants have been developed, Smart-Seq and Smart-Seq2, which differ in the 5’ end primer of the strand^[57,58]^. Later, Quartz-seq was developed to detect the heterogeneity of gene expression between groups of SCS methods. This method reduces the amplification to detect expression of genes in different single cells types^[59]^. Cell expression by linear amplification and sequencing (Cel-Seq and Cel-Seq2) uses the method of molecular barcoding to identify different single cells in a pool of cells^[60,61]^. Despite the cost-effectiveness of the technique, it remains under- development. Single cell tagged reverse transcription (STRT) is a type of sequencing that quantifies the 5’ mRNA gene expression in single cells, that is capable of locating promoters and enhancers. One of the latest is the Drop-Seq and Indrop-Seq by Islam *et al*.^[62]^ in which thousands of cells in a droplet are sequenced by using a wrapped unique barcode. Another method has been developed from fixed cells, and additional transcriptome and methylome analyses have been studied to determine changes in expression of RNA in single cells^[47,63]^. Several other variants are exemplified in Table 2 and reviewed in more details elsewhere.

Despite being a time-consuming technique that requires multiple sampling and cannot be used to make generalizations, SCS can be used to diagnose rare tumor cells, detect earlier metastatic malignancies in CTCs, and study intra-tumor heterogeneity^[50]^. Even though this technique provides high replicability can have a high generation of false-positive or negatives or sequencing bias, affecting the applicability of the technique to drug treatment and diagnosis. Understanding intra-tumor heterogeneity can help improve current cancer treatments through precision medicine. Take for example breast cancer, which has been classified as at least 18–21 subtypes with unique histological and molecular characteristics; yet therapy is delimited to the ER, PR, Her2 criteria^[79]^. Since intra-tumor heterogeneity leads to chemotherapy resistance^[79]^, SCS can help detect rare genotypes that may be an aid in this process. Intra-tumor heterogeneity may also confer some adaptive features to the tumor through distinctive biomarkers, so SCS can also help identify such biomarkers to improve current treatment selection and move forward into precise medicine.

## CENTROSOME ABERRATIONS, CHROMOSOME INSTABILITY AND TUMORIGENESIS

Over 100 years ago, Theodor Boveri coined the term centrosome (independently and simultaneously discovered and called corpuscle central by van Beneden) and hypothesized that centrosome aberrations leading to abnormal mitosis and abnormal chromosome constitutions may contribute to malignant tumors^[80]^. Since then, our laboratory and those of others have worked towards the elucidation of the mechanisms and consequences of centrosome aberrations in tumor initiation and progression. The centrosome is a small organelle composed of a pair of centrioles surrounded by pericentriolar material (PCM) that serves as the principal microtubule organizing center of vertebrate cells^[81]^. The centrosome duplicates only once to ensure proper spindle formation and equal chromosomal segregation during mitosis^[82,83]^. In order to maintain chromosome stability, the centrosome duplication cycle and the cell cycle must be tightly coordinated^[84–88]^. Laser ablation and microsurgical removal demonstrated that some immortalized mammalian cells (hTERTRPE and -HMECs) can cycle without centrioles/centrosomes; however, some epithelial cells like BSC-1 African green monkey kidney cells go through G1 much more slowly or not at all if centrosomes are removed^[89,90]^. Centrosome removal sensitizes cells to various external stimuli such as blue light, which results in p53-dependent G1 arrest^[89]^. Similarly, silencing of 14 (out of 15) centrosome components arrests cells in G1 by activating p53, p21, p38, and inactivation of cyclin A-Cdk2 activity^[91]^.

Failure in the control of the centrosome cycle or of cytokinesis leads to numerical and structural centrosome aberrations, which have been identified in most cancer types^[92–94]^. A common centrosome aberration in many cancers is centrosome amplification^[94]^, which culminates in different degrees of aneuploidy (including single chromosome gains/losses all the way to whole genome doubings) and chromosome instability, thus contributing to intra-tumor heterogeneity. In order to maintain genomic stability, the cell cycle machinery also regulates the centrosome cycle^[84,88,95–99]^. One model states that the centrosome duplication cycle starts in G1-S when the pair of centrioles dissociates^[88,100,101]^. In a model proposed by Fukasawa^[88]^, centrosome disengagement in late G1 is licensed by the phosphorylation of nucleophosmin (NPM) by cyclin E/Cdk2 complexes^[97,102–107]^. Another model, evidenced by data from the Stearn group suggests that centriole disengagement occurs during anaphase, that it involves separase, and that this event licenses centriole duplication in the next cell cycle; in this model Cdk2 is required for centriole duplication, but not for licensing^[108]^. Our studies added additional complexity to these models, since the centrosomes from *cdk4*^−/−^ mouse embryonic fibroblast did not achieve centrosome separation at G1/S, while these with a *cdk2*^−/−^ genotype achieved premature separation, and the premature separation defect was exacerbated in *cdk2*^−/−^*cdk4*^−/−^ mouse embryonic fibroblasts^[104]^. Early studies from the Nigg’s group demonstrated that centriole duplication requires the activation of E2F transcription factors and the activity of the Cdk2-cyclin A complex^[107]^, and the Leone laboratory demonstrated that repression by E2F3 played a major role in preventing premature centriole duplication, centrosome amplification, and chromosome instability by controlling cyclin E levels and cyclin E-dependent kinase activity^[109]^. Although it is not entirely clear how the E2F activators (E2F1, E2F2 and E2F3a) control centrosome duplication, our laboratory has shown that the E2F activators control the transcription, protein stability, and protein levels of many targets that regulate the centrosome cycle and mitosis, including cyclin E, Rb, Plk4, Nek2, Mps1, SgoL1, and cyclin B^[35,109,110]^.

**Figure 1.**
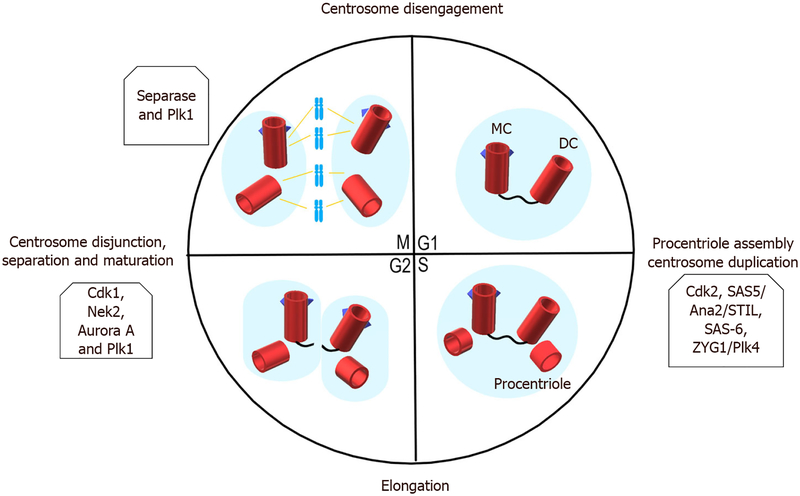
The centrosome duplication cycle. The mother centriole (MC) is depicted with blue triangles that represent the distal and sub-distal appendages to differentiate it from the daughter centriole (DC). In the G1 phase, the two centrioles are connected by a proteinaceous linker. The G1/S transition phase is characterized by the procentriole assembly, and some of the key proteins involved in this process are mentioned. In this stage, the DC starts to acquire the appendages that the MC has. During the S phase, the microtubules are synthesized, and rearrangement will occur to fully generate the procentriole. Till the G2 phase, the proteinaceous linker is broken, and the DC already has the distal and sub-distal appendages. This will convert DC into MC, and two pairs of centrioles will be formed. In the G2/M transition phase centrosome disjunction, separation, and maturation take place. Some key regulators have been listed above. During the M phase, the separated centrioles participate in bipolar spindle mitosis, and the centrosome cycle is completed when each daughter cell inherits two centrioles

Albeit elucidating the entire centrosome duplication cycle is still a work in progress, much is now known about the cellular events controlling it, recently reviewed by Nigg and Holland^[111]^. Centriole assembly is controlled by phosphorylation of Ana2/STIL by Plk4; this event recruits Ana2 and Sas6 to initiate procentriole formation^[112,113]^. Centriole biogenesis is controlled by interactions between Cdk2 and the SKP1-Cullin-F-box E3 ligase βTrCP, where Cdk2 protects STIL from degradation by βTrCP^[114]^; STIL then interacts with CPAP to complete centriole duplication^[115]^. Cdk2 also controls the degradation of Mps1 in centrosomes to control centriole duplication^[116]^. Aurora kinase A (AURKA) is essential to the formation of a bipolar mitotic spindle by regulating centrosome separation^[117]^. The AURKA phosphorylation of Cdk1-cyclin B at G2 recruits the former to centrosomes, where it is activated to initiate mitotic entry^[118]^. Centrosome localization of Cdk1 and inhibition of Chk1 is present in mitosis to prevent premature activation of the Cdk1-cyclin B complex^[119]^. Accordingly, PLK1 regulates centrosome maturation^[120]^, centrosome disjunction through NEK2^[121]^, and centrosome microtubule-attachments^[122]^. Also, NEK2 regulates centrosome separation by phosphorylating and inactivating c-Nap1 and β-catenin^[123,124]^. Lastly, from metaphase to anaphase, the two centrosomes migrate to opposite cellular poles and form the mitotic spindle to which the kinetochore will attach^[82]^. Faithful segregation of chromosomes is ensured by the spindle assembly checkpoint (SAC) and associated proteins such as BUB1B^[125]^, MPS1^[126]^, among others. Other proteins that play important functions in chromosome integrity include Bub1, which maintains sister chromatid cohesion through the phosphorylation of SgoI^[127]^; another protein that plays a key role in this activity is PP2A, which ensures localization of Sgo1 to centromeres^[128]^. Aurora kinase B, survivin, and ICENP play important roles in cytokinesis^[129]^ [Figure 1].

Deregulation of the centrosome duplication cycle results in centrosome aberrations and chromosome instability that ultimately have an effect on tumorigenesis^[87,88,130]^. While centrosome aberrations are traditionally associated with cancer, mutations in genes that codify for centrosome proteins are also known to cause human diseases such as ciliopathies (e.g., autosomal recessive primary microcephaly, Bardet-Biedl disease, polycystic kidney disease, and primary ciliary dyskinesia)^[131]^. Centrosome aberrations are classified as numerical and structural^[132]^. Both aberrations co-occur in tumors^[133,134]^. Centrosome aberrations have been identified in most cancer types^[94]^. For example, pioneering studies from the Doxsey laboratory demonstrated structural abnormalities in number, position, shape, and size of centrosomes in primary solid tumors, including brain, breast, colon, lung, and prostate^[92]^. Likewise, studies from the Salisbury laboratory showed that breast cancer tissue displayed abnormal structural and numerical centrosome aberrations, abnormal mitoses and chromosome instability relative to normal breast tissue^[133,135,136]^ and that centrosome amplification in breast cancers is indicative of tumor aggressiveness^[137]^. Centrosome amplification is defined as an excess of normal components, specifically more than two centrosomes and more than four centrioles^[138]^. Centrosome amplification results in multipolar or pseudobipolar mitotic spindles that may culminate in aneuploidy and chromosome instability^[101]^. Also, centrosome amplification may lead to defects in cytokinesis that lead to tetraploidy^[139]^. Because tetraploidy and excess chromosome instability are associated with decreased cellular fitness^[140,141]^, cells with amplified centrosomes avoid cell death by clustering centrosomes in order to avoid the generation of multipolar mitosis, and excessive aneuploidy and chromosome instability^[142,143]^. However, cells with pseudobipolar spindles form merotelic attachments that lead to single chromosome gains and losses^[144]^. Either tetraploidy or single chromosome losses have been shown to be tumorigenic in mouse models of cancer^[145,146]^. In a more recent study, Sabino *et al*.^[147]^ demonstrated that *Drosophila melanogaster* epithelial wing disc cells overexpressing Sak display extra centrosomes and exhibited mechanisms of clustering, but also inactivation of extra centrosomes. Inactivation of extra centrosomes is defined as the gradual loss of microtubulenucleating capacity. Although inactivation culminates in normal spindle bipolarization, neither clustering nor inactivation was efficient and abnormal segregation was observed. Furthermore, epithelial cells with extra centrosomes generated tumors when transplanted into the wild-type host.

Although the role of numerical aberrations (i.e., centrosome amplification) in cancer has been extensively studied, its role in tumor initiation, progression, and metastasis remains controversial, and may be context-dependent. For example, centrosome amplification in hepatobiliary cancer is not associated with tumor stage, size or proliferative activity^[94]^. Likewise, there is no significant relationship between centrosome amplification and tumor size, stage or patient survival in lung cancer^[94]^. Moreover, studies from the Cleveland group in mice - with centrosome amplification induced by Cre-recombinase-mediated Plk4 expression - did not result in spontaneous tumorigenesis regardless of p53 status^[148]^. Concordantly, studies from the Basto’s laboratory demonstrated that induction of centrosome amplification in mouse brains caused microcephaly due to increased apoptosis caused by multipolar divisions of neuronal stem cells^[149]^. Our own studies using an orthotopic model of breast cancer showed that rescuing back centrosome amplification in Her2^+^ breast cancer cells silenced for E2F3 through the overexpression of GFP-Nek2 did not influence tumor growth or tumor burden^[150]^. In contrast, other models suggest that centrosome amplification can influence tumor initiation and progression. For example, centrosome amplification correlates with poor prognostic factors such as nodal status and hormone receptor-negative status in 103 primary invasive breast cancers^[151]^. Likewise, centrosome amplification is associated with triple-negative breast cancers, higher stage, and higher grade, correlating with decreased overall survival and relapse-free survival in a cohort of 362 breast cancer patients^[152]^. Another study confirmed the above results and correlated centrosome amplification with markers of aggressiveness in triple-negative breast cancer patients, including increased stage and the mesenchymal marker vimentin^[153]^. Several transgenic models suggest that centrosome amplification might have causal, rather than consequential effects on cancer. For example, centrosome amplification causes tumors in flies independently of chromosome instability^[154,155]^. Other studies using transgenic mouse models involved the temporal expression of the prolyl isomerase Pin1^[156]^, Aurora A^[157]^, or K-Ras^G12D[25]^ in mammary epithelial cells, which resulted in pre-malignant mammary epithelial lesions with centrosome amplification that preceded mammary tumors. In mice, centrosome amplification induced by Plk4 accelerates the time of onset of lymphomas and sarcomas associated with loss of p53^[158]^, and of skin tumors in p53-deficient epidermis^[159]^. More recently, Levine *et al*.^[160]^ used a mouse model of intestinal neoplasia with a single truncated allele of the adenomatous polyposis coli (APC^Min^) tumor suppressor and generated a doxycycline-inducible mouse model exhibited increased levels of PLK4 (APC^Min/+^; Plk4^Dox^), which resulted in centrosome amplification and aneuploidy. Notably, the APC^Min/+^; Plk4^Dox^ exhibited higher intestinal tumor incidence compared to the APC^Min^ but no greater tumor burden. Therefore, these results demonstrate that centrosome amplification has a role in tumor initiation but not in tumor progression. To investigate if centrosome amplification can drive spontaneous tumorigenesis, Levine *et al*.^[160]^ also developed a ROSA26-*rtTA*; tetO-*Plk4* mouse model that expressed Plk4 in multiple mouse tissue upon doxycycline treatment. These mice developed lymphomas, squamous cell carcinomas, and sarcomas that exhibited aneuploidy. However, it is still unknown why some tissue efficiently develop tumors, where others do not. Perhaps this is due to the high levels of centrosome amplification induced in these models, since high-level chromosome instability and aneuploidy affect the fitness of tumor cells, since they die, or stop proliferating after a few cell cycles^[140,141]^.

Moreover, studies from the Pellman group demonstrated that centrosome amplification also plays a role in tumor progression by promoting invasion^[161]^. In this particular study, invasion was measured using a 3D culture model after inducing centrosome amplification in untransformed human mammary epithelial MCF10A cells either by a genetic approach (through the overexpression of PLK4 in the cells by a doxycyclineinducible system) or by a pharmacological approach (through the inhibition of cytokinesis by the addition of 1,4-Dichlorobenzene, DBC, which also resulted in tetraploidy)^[161]^. The advantages of using such approaches are that this model allows the visualization of invasive protrusions and breast glandular structure formation, which cannot be achieved by conventional cell culture. The major findings were that centrosome amplification induced invasion in breast cells through an increase in the activity of Rac1 that disrupted cell to cell adhesions, and the invasion was independent of the induction of tetraploidy^[161]^. Likewise, our laboratory showed that rescuing back centrosome amplification in Her2^+^ breast cancer cells downregulated for E2F3 by overexpressing GFP-Nek2 induced invasive protrusions in 3D culture^[162]^. The Aneja’s laboratory also showed that induction of centrosome amplification by overexpression of Plk4 in MCF10A cells induced higher migration that correlated with vimentin expression^[153]^. Experiments done by Denu expressing Plk4 in non-transformed MCF10A mammary epithelial cells demonstrated that acute acquisition of centrosome amplification resulted in de-differentiation of cells, where CD24 levels were reduced, and CD44 increased, suggesting that these cells were acquiring stem-cell features^[13]^.

While the role of centrosome amplification in cancer is more clearly defined, the role of structural aberrations has been unclear until recently. Structural centrosome aberrations are defined as changes in size and composition of the pericentriolar matrix without changes in the number of centrioles^[163]^. Overexpression of Ninein-like protein (Nlp), a protein that is involved in microtubule nucleation^[164]^ causes structural centrosome aberrations leading to spontaneous tumors in mice, including breast, ovary, and testicle^[165]^. The latest result from the Zhan laboratory is highly relevant to human disease since Nlp is overexpressed in breast, lung, ovarian, and squamous head and neck cancers^[165–167]^. Interestingly, structural centrosome aberrations lead to similar phenotypes as centrosome amplification, albeit by a non-cell autonomous mechanism, since overexpression of Nlp contributes to invasion by causing stiffness in epithelial cells that culminate in budding out of the acinar structures mitotic cells that do not contain centrosome aberrations^[168]^.

Together, these experiments suggest that centrosome amplification and structural aberrations can contribute to aggressive features of tumors by inducing invasion, increased grade/stage, and more stem-like features of cells. The studies above suggest that the effects of centrosome amplification in tumor cells appear to be context dependent.

## MECHANISMS DRIVING CENTROSOME AMPLIFICATION AND CHROMOSOME INSTABILITY

The Vande Woude group first identified the mechanism by which centrosome amplification is generated in tumors by showing that mouse embryonic fibroblasts lacking p53 displayed centrosome amplification^[169]^. Later on, other groups demonstrated that centrosome amplification was triggered by the loss of tumor suppressors that include APC^[170]^, BRCA1^[24]^, and BRCA2^[171]^. Regarding the mechanism, in p53-null mouse embryonic fibroblasts, silencing or genetic ablation of Cdk2 and Cdk4 suppressed centrosome amplification^[104]^. Also, centrosome amplification in Brca1- or GADD45- deficient cells was associated with the downregulation of Nek2^[172]^. Several studies revealed oncogenes could also drive centrosome amplification. For example, v-RAS drives centrosome amplification through the MAPK pathway^[26,173]^. Further, H-Ras^G12V^ and H-Ras^G12V^, and c-Myc drive centrosome amplification through cyclin D1, Cdk4, and Nek2 in the non-transformed mammary epithelial cells MCF10A^[25]^. Likewise, Her2^+^ breast cancer cells require Cdk4 and Nek2 to signal centrosome amplification and chromosome instability^[174]^. Further, the inhibition of Cdk2 suppressed Aurora A-induced centrosome amplification in MCF7 breast cancer cells with inactive p53 by preventing the localization of Aurora kinase A to centrosomes^[175]^. However, not all oncogenes induce centrosome amplification as means to initiate tumors, despite the induction of proliferation and apoptosis in pre-malignant mammary epithelial lesions by c-Myc; the pre-malignant lesions were devoid of centrosome amplification^[25]^. Nevertheless, c-Myc eventually induced centrosome amplification in mammary tumors, suggesting that c-Myc requires other genetic or epigenetic alterations to induce this abnormal process in mammary tumors.

There has been vast evidence demonstrating the essential role of the RB/E2F pathway in cell cycle regulation and centrosome duplication, a pathway that is unregulated by oncogenes such as Ras and Myc^[176]^. For example, acute loss of Rb causes centrosome amplification^[177]^. Although the E2F transcriptional factors have redundant functions, each member of the family also has unique functions^[178]^. Take for example E2F3, whose loss in mouse embryonic fibroblasts results in unregulated cyclin E-dependent kinase activity, defects in nucleophosmin B association with centrosomes, and premature centriole separation and duplication that result in centrosome amplification, mitotic spindle defects, and aneuploidy^[109]^. On the other hand, genetic ablation of E2F1, E2F2, E2F4 or E2F5 does not cause centrosome amplification. Also, silencing E2F1 or E2F3 in Her2^+^ breast cancer cells suppresses centrosome amplification, while overexpression of E2F1, E2F2, or E2F3a in MCF10A cells is sufficient to trigger centrosome amplification and chromosome instability^[110]^.

Chromosome instability is a broad term that refers to chromosome segregation errors, which results in chromosome losses or rearrangements. As reviewed elsewhere, chromosome instability can occur as a consequence of mitotic checkpoint defects, aberrations in centrosome duplication cycle, altered kinetochore function, microtubule attachment defects, chromosome cohesion defects, and mutations causing or allowing genomic instability^[17]^. Although it has been shown that centrosome amplification leads to chromosome instability^[101]^, a recent study from Kuznetsova *et al*.^[179]^ showed that chromosome instability, tolerance of mitotic errors, and multidrug resistance can be promoted by tetraploidization in human cells without centrosome amplification. This study demonstrated that chromosome instability was tolerated by mutations in p53 and the downregulation of the pro-apoptotic factors iASPP and cIAP2. Even though it remains a question whether centrosome amplification is a cause or an effect of chromosome instability, both have been shown to occur exclusively in malignant tumors that display aneuploidy^[138]^ and are associated with tumor recurrence^[180]^, metastasis^[181,182]^, and drug resistance^[18,183,184]^. Aneuploidy is defined by gains or losses of whole chromosomes that play a role in tumor initiation, maintenance, and progression^[138]^. Aneuploidy, as a consequence of chromosomal instability, along with genomic instability (defects in DNA damage detection and repair) lead to intra-tumor heterogeneity.

Chromosome instability occurs exclusively in malignant tumors that display aneuploidy; chromosome instability affects tumor progression by generating intra-tumor heterogeneity^[181,182]^. For example, chromosome instability has been shown to maintain intra-tumor heterogeneity in glioma cells^[185]^. A more recent study showed that chromosome missegregation drives intra-tumor heterogeneity in glioma cells; cells with double minute chromosomes were more radio-resistant than those without them^[186]^. Upon irradiation, the double minute chromosomes allowed glioma cells to invade and become angiogenic. Thus, in that setting, intra-tumor heterogeneity generated by the loss and gains of double minute chromosomes may hinder cancer treatment by increasing cell invasiveness and radio-resistant cells. Several studies have shown that chromosome instability also contributes to chemotherapy resistance^[18,183,184,187]^, making chromosome instability a good therapeutic target. However, it is noteworthy that there is a complex relationship between chromosome instability and therapeutic response that depends not only on the chromosome instability level, but also in the genetic context and tissue type^[188]^. As an example, a study conducted by Heerema *et al*.^[188]^ found that trisomies of chromosome 4 and 6 did not affect prognosis in patients with high hyperdiploid acute lymphoblastic leukemia, while concurrent trisomies of chromosomes 10 and 17 were associated with a better prognosis and trisomies of chromosome 5 was correlated with a worse prognosis. Later on, in this manuscript, we describe two approaches to target chromosome instability clinically. The first is by targeting some key proteins involved in the centrosome duplication cycle to decrease chromosome instability. The second approach aligns more with the notion that the cell will tolerate a certain level of chromosome instability and beyond that the cell will not be viable. Therefore, this approach aims to elevate chromosome instability levels to induce cell cycle arrest or apoptosis.

## INHIBITORS OF CHROMOSOME INSTABILITY IN CANCER TREATMENT

Given the numerous mechanisms attributed to chromosomal instability, several approaches have been proposed to target chromosome instability in cancer. One approach is to target centrosome-associated proteins that regulate microtubule dynamics and the SAC to prevent centrosome amplification, thus preventing chromosome instability^[87,189,190]^. The Cdk4/Cdk6 inhibitor Palbociclib (PD-0332991) in combination with the aromatase inhibitor letrozole has greatly improved the outcomes of ER^+^, Her2^−^ advanced breast cancer patients^[191,192]^. Albeit that study did not measure centrosome amplification and chromosome instability, it is tempting to propose this as an approach to suppress active generation of these processes in cancer cells, since we have shown that silencing or genetic ablation of Cdk4 in p53-null fibroblasts, in mammary epithelial cells expressing H-Ras^G12V^ or H-Ras^G12V^ and c-Myc, or in Her2^+^ breast cancer cells suppress these processes^[25,104,174]^. However, this approach neglects the fact that chromosome instability may occur by multiple mechanisms and multiple dysregulated proteins. In fact, Palbociclib is ineffective in basal breast cancer cells (the subtype with a higher degree of chromosome instability), and patients are harboring alterations in the Rb/E2F pathway^[193,194]^. Nevertheless, several inhibitors targeting polo-like kinases (Plks) and Aurora kinases (AURKs) have been tested in pre-clinical and clinical trials with mixed outcomes, and this has been extensively discussed elsewhere^[189]^. Notably, the inhibitor MLN8237 (Asertib) that targets AURKs exhibited efficacy for several solid tumors and T-cell lymphoma, but not acute myeloid leukemia^[195,196]^. The opposite was observed for the selective inhibitor of AURKB, AZD1152 (Barasertib)^[189]^.

Another strategy to kill tumor cells is to elevate chromosome mis-segregation. It has been proposed that there is an optimal level of chromosome instability for tumor maintenance and progression; beyond that level chromosome instability becomes detrimental for cancer cells^[12,184]^. For example, elegant experiments from the Sluder laboratory demonstrated that the acquisition of tetraploidy in most immortalized or cancer cells they investigated resulted in cell cycle arrest within a few cell cycles^[140]^. Also, the Cleveland group demonstrated that while low-level aneuploidy triggered by the loss of one copy of Cenp-E was tumor promoting in mice, aneuploidy can also be tumor-suppressive^[197]^. A recent pan-cancer analysis of genetic heterogeneity in cancer done by the Malley group showed that in general, cancers with intermediate levels of chromosome instability (measured by copy number variation analysis) had worst prognosis than cancers with low or high levels of instability^[2]^. However, their relationship varied depending of the adjuvant treatment given, suggesting that radiotherapy and adjuvant chemotherapy may be effective in treating cancers with intermediate chromosome instability by pushing the limits of tolerable chromosome instability. The Swanton’s group also provided clinical evidence to support this hypothesis with their retrospective study conducted in a cohort of 246 primary breast cancer patients^[12]^. The study showed that extreme chromosome instability (measured with chromosome-specific markers and aCGH and correlated to the CIN70 score, MammaPrint, and GGI) correlated with improved long-term survival in ER-negative breast cancer patients; exhibiting a non-monotonic correlation^[12]^. This observation was confirmed in a study involving a larger cohort of ER^−^ patients^[198]^. However, a linear correlation was observed in ER-positive breast cancer patients and extreme chromosome instability^[12]^; the same relationship was found with glioblastomas^[2]^. Thus, we have to be careful with proposing increasing chromosome instability as a strategy against cancer, since it is tumor suppressive in some cancers, and tumor promoting in others.

Mitotic kinases contribute to chemotherapy resistance, as illustrated by Janssen *et al*.^[199]^, who demonstrated that the reduction of essential levels of Mps1 and BubR1 sensitized several tumor cells to clinically relevant doses of paclitaxel (an anti-mitotic drug commonly used in cancer treatment). On the other hand, inhibition of these kinases did not induce cell death in normal cells. Currently, a Mps1 inhibitor is being tested in clinical trial Phase 1 (BAY1161909) in triple negative breast cancer patients^[200]^. In this clinical trial, the Mps1 inhibitor is administered along with paclitaxel (a microtubule-interfering agent) to induce tumor death by increased chromosome mis-segregation^[200]^. A similar approach can be tested with the combination of paclitaxel and BubR1, Hec1, Nek2, or Sgol1 inhibitors because all of these proteins play an important role in proper SAC functioning and our studies have demonstrated their role in centrosome amplification and chromosome instability downstream of the E2F activators^[35,162,201]^. Additionally, a study by Lee *et al*.^[201]^ ranked 62 different anticancer drugs for their capacity to induce chromosome instability. The drugs evaluated in this study have several mechanisms of action (e.g., antimicrotubule activity, DNA replication and damage response, mitotic checkpoint inhibition, *etc*.) and can be evaluated in combination with inhibitors of centrosome-associated proteins to see if the effect of increase chromosome instability is potentiated. Thus, these findings present us with multiple possibilities that together with advances in precise medicine and technologies such as SCS can be explored in cancer patients with specific tumor genotype/phenotype (intra-tumor heterogeneity) to develop better treatment.

## CONCLUSION

**Figure 2.**
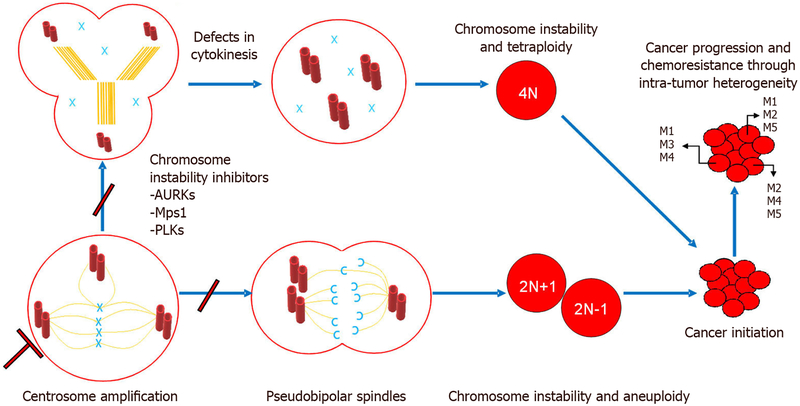
Centrosome amplification leads to tumor initiation and cancer progression through intra-tumor heterogeneity. Two models are described above. First, centrosome amplification leads to pseudobipolar spindles that culminate in chromosome instability and aneuploidy. Second, centrosome amplification leads to defects in cytokinesis that culminates in chromosome instability and tetraploidy. Both mechanisms converge to initiate cancer. Cancer progression and chemoresistance occurs and is maintained as a consequence of intra-tumor heterogeneity. Chromosome instability inhibitors (e.g., AURKs, Mps1, and PLKs) are therapeutic targets that may prevent this chain of events by targeting early steps of this process

Failure to properly regulate the cell cycle and the centrosome cycle leads to centrosome aberrations. One of such centrosome aberrations is centrosome amplification, which occurs in various cancer types. In our model depicted in Figure 2, we summarize two known mechanisms that denote the role of centrosome amplification in tumor initiation, maintenance, progression, and chemo/radio-resistance through intra-tumor heterogeneity. One mechanism shows that centrosome amplification results in multipolar or pseudobipolar mitotic spindles that may culminate in aneuploidy and chromosome instability, thus contributing to intra-tumor heterogeneity. The other mechanism shows how defects in cytokinesis lead to tetraploidy and chromosome instability. This mechanism also promotes tumor initiation, maintenance, progression, and chemoresistance through intra-tumor heterogeneity. The reader should also keep in mind that centrosome aberrations may contribute to malignant phenotypes in cancer such as invasion through changes in polarity, and such phenotypes occur independently of chromosome instability.

However, the role of centrosome amplification in tumorigenesis needs to be further elucidated in human tumors because it has been shown that centrosome aberrations are highly context-dependent and several other mechanisms may apply^[202]^. Another aspect that is worth studying in the future is the effect of functional centrosome aberrations (microtubule nucleation, disorganized mitotic spindle, *etc*.) and other structural centrosome aberrations such as changes in shape, size position, and composition in cancer. Also, clustering mechanisms and normal spindle bipolarization through extra chromosome inactivation and how these vary in cancer. Nevertheless, proper classification of centrosome aberrations in human tumors might have a diagnostic or prognostic value. Therefore, it would be beneficial to explore the therapeutic applications of chromosome instability in cancer. As reviewed here, chromosome instability inhibitors such as AURKs, Mps1, and PLKs inhibitors can help improve cancer treatment by preventing centrosome amplification and chromosome instability. Another strategy will be to increase chromosome instability levels to promote cancer cell death, but this will be context dependent. For example, this strategy can be used for ER^−^ breast cancers, since extreme chromosome instability correlates with better prognosis in patients with this molecular phenotypes. On the other hand, increasing chromosome instability in ER^+^ breast tumors is a poor strategy, since there is a direct relationship between increases in chromosome instability and poor survival. In addition, increasing chromosome instability may increase chemotherapy resistance in some patients. SCS can help to address specific genotype that confers cancer cell subpopulations adaptive advantages and impede complete tumor clearance. The advances in both SCS and the identification of putative therapeutic targets are promising toward a complete understanding of cancer and how effective treatment can be achieved.

## Figures and Tables

**Table 1.** Single-cell genomic sequencing methods

Technique	Description	References
DOP-PCR	Allows the amplification of the nucleus genome using primers with ACTG combinations	[52]
MDA	No PCR phase; instead denaturalized DNA is amplified	[53–55]
MALBAC	Detects Copy Number Variants by amplifying the original DNA strand	[53]

**Table 2.** Single-cell RNA sequencing methods

Methods	Description	References
scRNA-Seq	Single cell transcriptome analysis	[56]
STRT-Seq	Provides adaptation of the template by switching oligonucleotide to barcode the 5’ of the transcripts; allows for unbiased amplification among samples	[62]
Smart-Seq	Allows the evaluation of single nucleotide polymorphisms in a full length of cDNA to barcode 96 samples	[58]
Cel-Seq	Single cell *in vitro* technique that amplified mRNA linear that was multiplexed in a barcode manner	[60,61]
Smart-Seq2	Improved the sensitivity, coverage, and accuracy using an inaccessible RNA nucleotide (locked nucleic acid)	[57]
RCA	Whole transcriptome amplification from a small quantity of DNA	[64]
FISSEQ	*In situ* whole transcriptome amplification from a small quantity of DNA	[65]
UMI	Unique molecule identifiers that are tagged to cDNA allows for adjusted amplification bias, sensitivity, and background noise of samples	[66]
Microfluidics	96-single cell Smart-Seq2 that uses a microfluidic system	[67]
inDrop-Seq	Droplet-based; allows the sampling of thousands of cells to be sequenced with a barcode wrapped	[68]
Drop-Seq	droplet	[69]
Cyto-Seq	Uses magnetic beads in combination with capture and poly(A) selection to analyze 100,000 cells	[70]
SUPeR-Seq	Uses a universal poly(A) independent RNA sequencing	[71]
G&T-Seq	Simultaneous genome and transcriptome sequencing	[72]
FRISCR-Seq	Uses intracellular staining; contains a low degree of bias	[73]
scMT-Seq	Simultaneously analyzes the methylome and the transcriptome of single cells	[74]
scTrio-Seq	Simultaneously sequence the genomic, transcriptomic, and methylome of single cells	[75]
Div-Seq	Scalable single nucleus RNA sequencing (sNuc-Seq), based that tracks dynamics of cells with high sensitivity	[76]
LCM-Seq	Laser capture microdissection *in situ* RNA sequencing	[77]
Small RNA-Seq	Analysis of micro, small, and transference RNAs	[78]
